# Effective and Lifesaving Retrograde Femoral and Jugular Vein Double‐Lumen Catheter Insertion in Patients With Poor Hemodialysis Venous Access Who Needed Emergency Hemodialysis: A Case Series

**DOI:** 10.1002/ccr3.70522

**Published:** 2025-05-19

**Authors:** Ahmad Hosseinzadeh, Farzad Dalfardi, Reza Shahriarirad, Fatemehsadat Pezeshkian, Farhad Keikha

**Affiliations:** ^1^ Thoracic and Vascular Surgery Research Center Shiraz University of Medical Sciences Shiraz Iran; ^2^ Student Research Committee Shiraz University of Medical Sciences Shiraz Iran; ^3^ School of Medicine Shiraz University of Medical Sciences Shiraz Iran

**Keywords:** emergency hemodialysis, resource‐limited settings, retrograde femoral catheterization, temporary dialysis access, vascular access

## Abstract

Retrograde femoral catheter insertion for hemodialysis access is an immediate rescue procedure and a short‐term solution to salvage life in compromised patients. This temporary solution could be reserved in resource‐limited regions until permanent dialysis routes are established.

**Abbreviations**
AVF: Arteriovenous fistula
AVG: Arteriovenous graft
BUN: Blood urea nitrogen
CAPD: Continuous ambulatory peritoneal dialysis
CDS: Color Doppler sonography
Cr: Creatinine
CVC: Central venous catheters
DVT: Deep vein thrombosis
ESRD: End‐stage renal disease
HD: Hemodialysis
IVC: Inferior vena cava
IVC: Intensive care unit
K: Potassium
Na: Sodium

## 1 Introduction

Central venous catheters are utilized to offer access for hemodialysis (HD) in individuals who need dialysis initiation or end‐stage renal disease (ESRD) patients who are awaiting permanent vascular access maturation, such as arteriovenous fistula (AVF), or less popularly, arteriovenous graft (AVG) [1]. Their safety and accessibility made them universally applicable and a straightforward access technique. However, central vein stenosis and occlusion due to infection and thrombosis will result in lower blood flow, longer dialysis sessions, and low patient satisfaction [2, 3].

Traditionally, the right jugular vein is approached first Subsequently, the right external and left jugular veins are approached afterward in case of occlusion and venous exhaustion—alternatively, femoral, translumbar, and further specialized access points such as transhepatic and transrenal veins. As the arms would instead be saved for future AVF or AVG plans, the subclavian vein is spared from catheter insertion and should be saved as a last resort [1, 4].

A novel approach was presented for individuals with challenging central venous access. This novel technique cannulates the superficial femoral vein by inserting a noncuffed HD catheter and further leading the guide wire and catheter retrogradely toward the distal part of the limb [5].

In the present study, we discuss two cases with previously failed traditional methods for central venous catheters that underwent retrograde femoral vein and left jugular vein catheter insertion successfully used for HD when the patients needed venous access emergently for uremia toxicity and electrolyte imbalance.

## 2 Case 1. History/Examination

A 67‐year‐old man who was diagnosed with ESRD and was put on routine HD in the past 5 years with no other comorbidities. He underwent routine HD three times a week. In the previous 5 years, he had undergone several central vein cuffed catheter insertions via the jugular, subclavian, and femoral veins. However, due to his latest femoral vein catheter dysfunction, he was referred to our referral center at Namazi Hospital, Shiraz, Iran, for CVC insertion. He had missed his dialysis sessions during the past month. He was admitted with a blood urea nitrogen (BUN) of 90 mg/dL, creatinine (Cr) of 16.2, sodium (Na) of 136 mEq/L, and potassium (K) of 5.7 mEq/L.

Venography through central venous puncture demonstrated bilateral brachiocephalic and internal jugular vein occlusion. Furthermore, the femoral vein catheter could not be inserted due to bilateral iliac vein occlusion, resulting from previously inserted catheters. A temporary double‐lumen catheter was inserted in his subclavian vein, which was subsequently compromised the following week. Interventional radiologists were not triumphant in translumbar vena cava catheter insertion. As he developed uremic encephalopathy and hyperkalemia, he was transferred to the operating room to unearth an accessible central vein for HD, as his clinical status did not allow proper time for arteriovenous fistula/graft and arterioarterial grafts.

## 3 Case 1. Conclusion and Results

**FIGURE 1:**
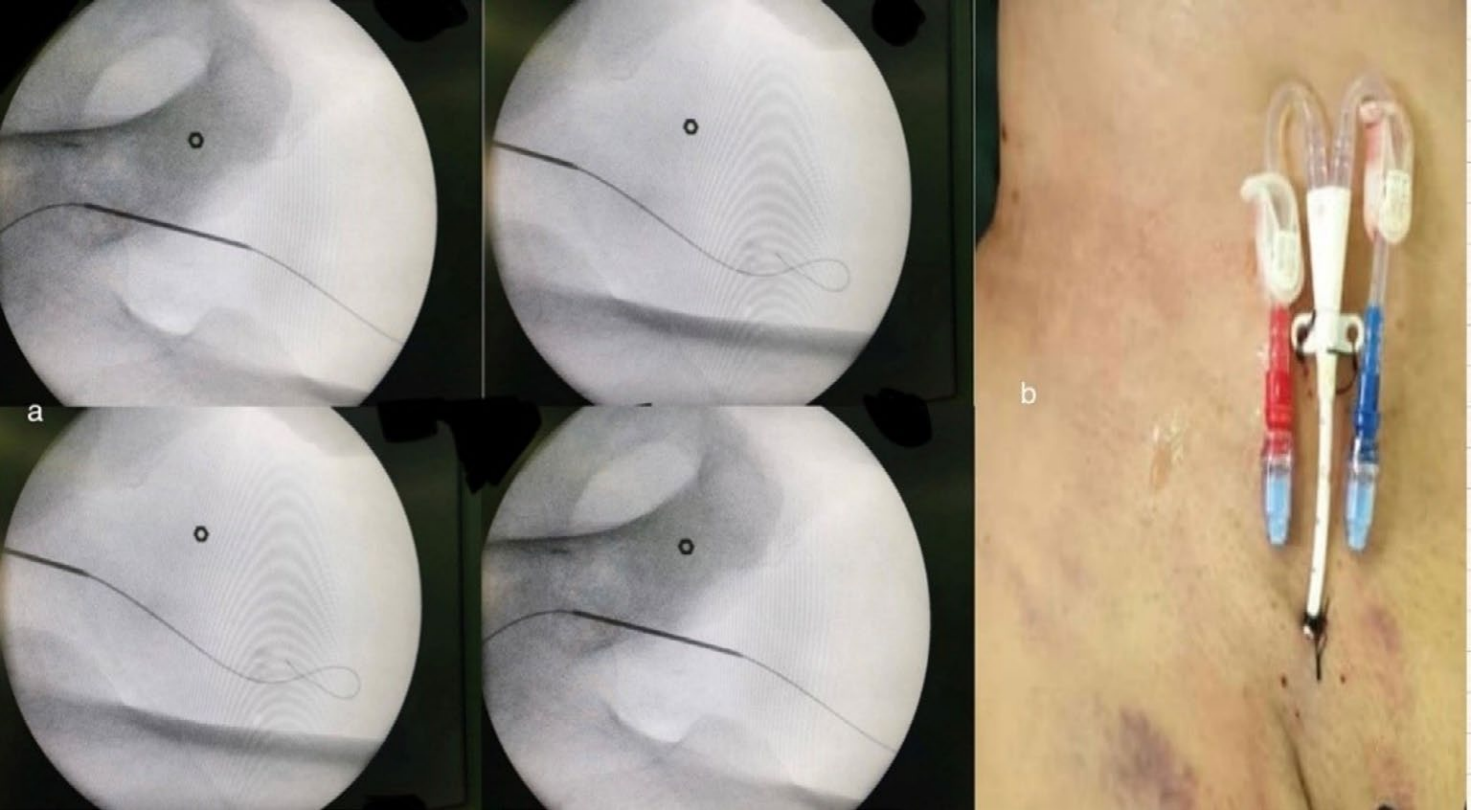
(a) Fluoroscopy showing a temporary hemodialysis catheter inserted retrogradely in the left superficial femoral vein by Slendinger's maneuver, (b) Retrograde superficial femoral vein catheter, secured with sutures and a dressing applied, located in the upper thigh and faced downward.

Under the sonography guide, his deep femoral vein was observed to have proper dilatation for catheter insertion. A needle was guided retrogradely into the common femoral vein, and the hydrophilic guide wire was then led into the vein access. Consequent venography indicated that the guide wire was within the deep femoral vein. The catheter was then retrogradely placed in the deep femoral vein with the Seldinger maneuver and was checked afterward, demonstrated proper blood flow, and was heparinized and fixed (Figure 1). Later, the patient underwent 2 h of HD successfully. The HD settings were Kt/v = 0.75 with bicarbonate as the buffer, a heparin bolus of 5000 cc, and 1500 cc ultrafiltration. The catheter was flushed with heparin 5000 units every 8 h and locked with a heparinate device. The post‐HD lab data of BUN: 41 mg/dL, Cr: 7.54 mg/dL, Na: 135 mEq/L, and K: 5.6 mEq/L were satisfied with the results without any catheter‐related adverse event. Table 1 summarizes pre‐ and post‐HD lab results. In his follow‐up, the patient underwent transhepatic catheter insertion by an interventional radiologist, which was functional for 2 weeks. The retrograde catheter in the femoral vein was discontinued 2 weeks later. Afterward, the transhepatic catheter was dysfunctional again, and another retrograde femoral catheter was inserted for him to buy time for CAPD catheter insertion and peritoneal dialysis.

**TABLE 1:** Lab report summary of pre‐and post‐hemodialysis treatment of patients.

Lab variables	Case 1		Case 2	
	Pre‐hemodialysis	Post‐hemodialysis	Pre‐hemodialysis	Post‐hemodialysis
Blood urea nitrogen (mg/dL)	90	41	42	22
Creatinine (mg/dL)	16.2	7.54	11.63	5.82
Sodium (mEq/L)	136	135	123	134
Potassium (mEq/L)	5.7	5.6	6.6	4.3
pH	7.39	7.38	7.38	7.44
PCO2 (mmHg)	29	32	26.6	30.2
Bicarbonate (mEq/L)	17.6	18.8	15.9	20.4

## 4 Case 2. History/Examination

A 68‐year‐old woman with a history of ESRD for the past 12 years, diabetes, and hypertension status post bilateral nephrectomy and kidney transplantwas referred to Namazi Hospital due to poor central venous access for HD. Physical examination revealed rales in both lung fields and evidence of pulmonary edema. Her lab data revealed BUN of 42 mg/dL, Cr of 11.63 mg/dL, Na of 123 mEq/L, and K of 6.6 mEq/L.

Her transhepatic catheter was dysfunctional, and interventional radiologists were unsuccessful in inserting a new translumbar vena cava catheter. Venography revealed that the inferior vena cava (IVC), brachiocephalic, jugular, iliac, and femoral veins were all occluded due to previous catheter and deep vein thrombosis (DVT) events in both thighs. Interventional radiologists were not successful in inserting a new hepatic vein catheter. Previous frequent surgeries in her abdominal area and the hematoma that was an adverse effect of radiologic intervention excluded peritoneal dialysis as a route for HD. Due to her missed HD sessions, her condition deteriorated, and she had resistant hyperkalemia (K: 6.6 mEq/L) and was later intubated due to pulmonary edema; all in all, her clinical status did not allow the time needed for arterioarterial graft maturation, and she had to go to the operation room to obtain venous access for HD.

## 5 Case 2. Conclusion and Results

**FIGURE 2:**
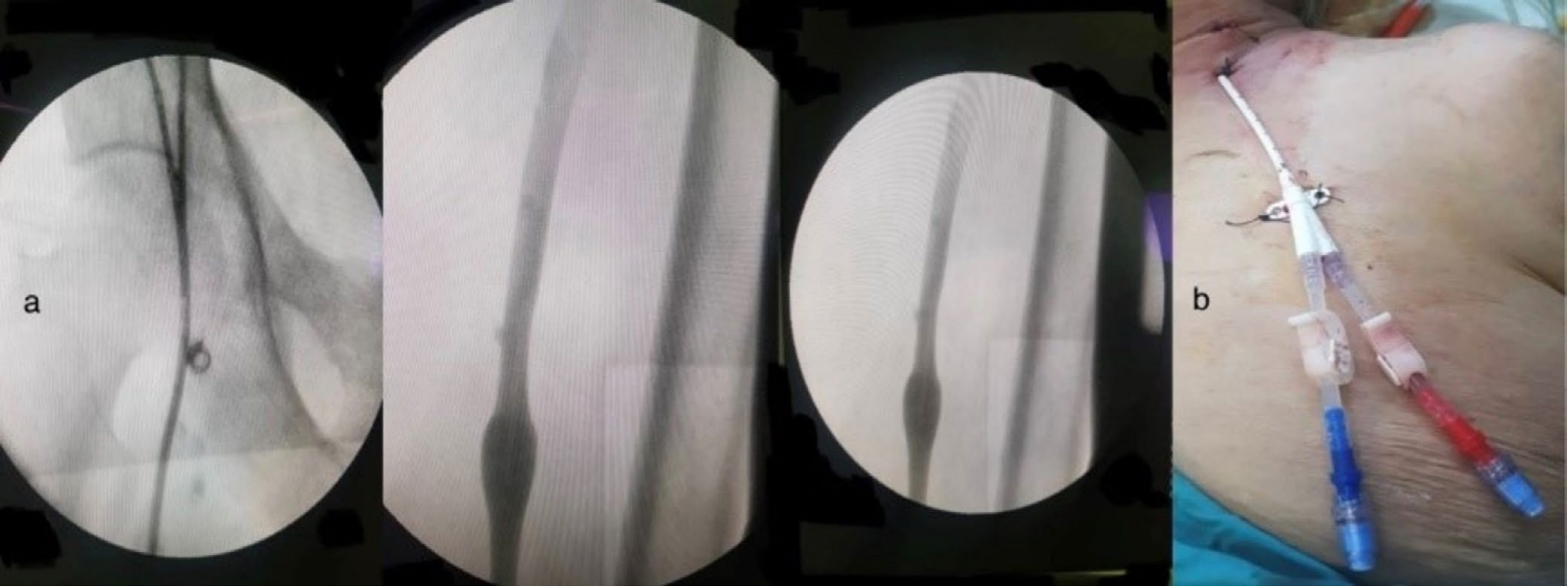
(a) Fluoroscopy showing a temporary hemodialysis catheter inserted retrogradely in the left jugular vein, (b) Retrograde jugular vein catheter, secured with sutures and a dressing applied, located on patient's lower neck area and faced downward.

Initially, all candidate central veins were assessed by sonography, and the only accessible vessel was her left jugular vein. A 12f double‐lumen catheter was retrogradely inserted under the sonography guide, and c‐arm fluoroscopy was used to determine the proper placement of the catheter (Figure 2). Afterward, the patient was transferred to the intensive care unit (ICU) and successfully underwent three HD sessions. The 4 h HD was carried out with a blood flow rate of 250 cc/min, dialysis flow rate of 500 cc/min, bicarbonate as the HD buffer, 1500 cc of ultrafiltration, Kt/v = 0.75, and a heparin bolus of 2500 cc. Her pre‐HD and post‐HD blood pressures were 125/72 mmHg and 102/48 mmHg, respectively. She was extubated with the lab data of BUN: 36 mg/dL, Cr: 10.4 mg/dL, Na: 130 mEq/L, and K: 4.8 mEq/L. Detailed pre‐ and post‐HD lab data are summarized in Table 1. In 3 weeks, she underwent 10 sessions of HD successfully. She was satisfied with the procedure; the catheter was functional without any catheter‐related adverse events, and the left axillary artery interposition loop goretex arterioarterial graft was scheduled for her.

## 6 Discussion

Retrograde femoral vein catheter insertion is reserved for emergent, life‐threatening conditions in patients for whom other traditional HD access ways were not feasible, such as internal jugular vein occlusion [6]. However, it should be noted that this is a salvageable option that is most relevant in resource‐limited regions and can act as a temporary HD route until permanent access is established. The dilatation of the superficial femoral vein and valvular dysfunction, secondary to common femoral and/or external iliac vein stenosis or obstruction, allows the guidewire to pass in a retrograde direction toward the foot. In these patients, the femoral vein dilates from its average width of 12 mm to 14–16 mm, allowing easy insertion of a non‐cuffed 4 mm catheter [5].

The superficial femoral vein was deemed a safe route for CVC insertion without any severe risk of complications due to insertion [7]. Inserting a retrograde femoral catheter is feasible in any setting and equipment without special training and skills. It is mandatory to obtain a complete physical examination and history to rule out the possibility of DVT prior to catheter insertion. Color Doppler sonography (CDS) assesses the femoral vein width and its competency and ensures the catheter is inserted correctly. However, the femoral vein can be punctured blindly if CDS is out of reach or the patient's condition is emergent and life‐threatening [5].

This approach is advantageous for the selected patients with continuous ambulatory peritoneal dialysis (CAPD) with poor venous access and malfunctioning Tenchoff catheters needing an emergent HD and not having enough time for catheter replacement or manipulation [5].

Retrograde femoral vein catheter was observed to alleviate the patients' uremia and hyperkalemia state in our study. A retrospective inspection of six patients who presented with poor vascular access for HD and had undergone retrograde femoral vein catheter insertion with the Slendinger maneuver revealed that this approach was beneficial in urgent conditions [5, 8]. Their HD sessions were of acceptable quality, and it was triumphant in solving patients' hyperkalemia and uremic state in HD. The catheter was functional for an average of about 3 days and three successful HD sessions. This borrowed time was utilized for CAPD catheter manipulation or inserting a cuffed catheter [5]. All in all, inserting retrograde femoral vein catheters can be considered a time‐saving technique to reconsider more permanent methods for HD access in patients with poor HD access.

The mentioned study took short‐term complications into account. Mild blood ooze was observed from the site of insertion, which was controlled by applying mild compression to the site, which is an expected complication in all methods of CVC insertion [9]. Moreover, mild discomfort was also reported, which is expected in all femoral catheters. The catheter of one of the participants had to be removed due to the pain during the HD session. DVT or sepsis was not reported in the six patients due to the short number of catheter days. However, the mentioned study did not follow the patients after discharge and did not include long‐term complications.

In conclusion, this alternative technique of retrograde CVC insertion could potentially be a lifesaving and time‐saving temporary method for patients whose previous conventional methods have failed. This technique will build a bridge that the physicians need to plan a more permanent HD route. It should be noted that this technique is more relevant to practices in resource‐limited regions. Further clinical guidelines and assessments are required to establish a safe guideline for inserting CVC retrogradely and to assess its function over time and possible side effects compared to conventional routes.

## Author Contributions

**Ahmad Hosseinzadeh:** conceptualization, methodology, project administration, resources, supervision, validation, writing – review and editing. **Farzad Dalfardi:** investigation, project administration, visualization, writing – review and editing. **Reza Shahriarirad:** data curation, methodology, visualization, writing – review and editing. **Fatemehsadat Pezeshkian:** investigation, methodology, visualization, writing – original draft, writing – review and editing. **Farhad Keikha:** investigation, project administration, resources, writing – review and editing.

## Ethics Statement

Ethical approval of the study and written informed consent were obtained from the patients in our study. The purpose of this research was thoroughly explained to the patients, and they were assured that the researcher would keep their information confidential. The present study was approved by the Medical Ethics Committee of the academy.

## Consent

Written informed consent for publication of their clinical details and/or clinical images was obtained from the patients. A copy of the consent form is available for review by the Editor of this journal.

## Conflicts of Interest

The authors declare no conflicts of interest.

## Data Availability

The findings of the present study are available on request from the corresponding author. They are not publicly available due to privacy and ethical restrictions.
